# Comparative proteomic analysis of a membrane-enriched fraction from flag leaves reveals responses to chemical hybridization agent SQ-1 in wheat

**DOI:** 10.3389/fpls.2015.00669

**Published:** 2015-08-26

**Authors:** Qilu Song, Shuping Wang, Gaisheng Zhang, Ying Li, Zheng Li, Jialin Guo, Na Niu, Junwei Wang, Shoucai Ma

**Affiliations:** College of Agronomy, Northwest Agriculture and Forestry University, National Yangling Agricultural Biotechnology and Breeding Center, Yangling Branch of State Wheat Improvement Centre, Wheat Breeding Engineering Research Center, Ministry of Education, Key Laboratory of Crop Heterosis of Shaanxi ProvinceYangling, China

**Keywords:** wheat, flag leaves, membrane proteome, CHA-SQ-1, ROS

## Abstract

The induction of wheat male fertile lines by using the chemical hybridizing agent SQ-1 (CHA-SQ-1) is an effective approach in the utilization of heterosis; however, the molecular basis of male fertility remains unknown. Wheat flag leaves are the initial receptors of CHA-SQ-1 and their membrane structure plays a vital role in response to CHA-SQ-1 stress. To investigate the response of wheat flag leaves to CHA-SQ-1 stress, we compared their quantitative proteomic profiles in the absence and presence of CHA-SQ-1. Our results indicated that wheat flag leaves suffered oxidative stress during CHA-SQ-1 treatments. Leaf O_2_^-^, H_2_O_2_, and malonaldehyde levels were significantly increased within 10 h after CHA-SQ-1 treatment, while the activities of major antioxidant enzymes such as superoxide dismutase, catalase, and guaiacol peroxidase were significantly reduced. Proteome profiles of membrane-enriched fraction showed a change in the abundance of a battery of membrane proteins involved in multiple biological processes. These variable proteins mainly impaired photosynthesis, ATP synthesis protein mechanisms and were involved in the response to stress. These results provide an explanation of the relationships between membrane proteomes and anther abortion and the practical application of CHA for hybrid breeding.

## Introduction

Membranes are highly organized structures and important components of plant cells. The membrane system of plant cells includes the plasma membrane (PM) and organelle membranes (nuclear membrane, endoplasmic reticulum (ER) membrane, Golgi membrane, mitochondrial membrane, chloroplast membrane, and lysosomal membrane). Membranes not only form cellular compartments for performing multiple differential metabolic processes and maintaining organelle homeostasis, but they also play a critical role in the exchange of substances and signals (Jaiswal et al., 2012). Lipids and proteins are major components of membranes. Membrane proteins take part in multiple biological reactions such as metabolite and ion exchange, signal transduction, biosynthesis, photosynthesis, and energy generation (Kota and Goshe, 2011; Liu et al., 2011; Takahashi et al., 2013).

Plants may suffer various biotic and abiotic stresses during growth, which can cause damage to cellular functions. However, within a certain threshold, plants have a battery of protective mechanisms to maintain the stability of the normal mechanical processes of cellular homeostasis. These protective mechanisms include changes in lipid composition and regulation of protein expression. Some reports show that changes in the ratio of lipid composition and an increase in the level of unsaturation of membrane lipids in some species under various stressors are effective in maintaining a stable membrane conformation (Welti et al., 2002; Gigon et al., 2004; López-Pérez et al., 2009). Membrane proteins play a vital role in plant stress response. Nouri and Komatsu (2010) suggested that transcription and expression of H^+^-ATPase in *Arabidopsis thaliana* are increased, which then accelerates the transport of ions between the inner and outer PM to maintain cellular homeostasis during hyperosmotic stress. Some studies have indicated that proteins of photosystem II (PS II), including chlorophyll a/b binding protein, ATP generating proteins, and NAD(P)H-quinone oxidoreductase, are up-regulated in *Aeluropus lagopoides*, barley, and mangrove plants under salt stress, which can facilitate photon capture, provide sufficient energy, detoxify quinines and maintain normal photosynthesis process (Sobhanian et al., 2010; Rasoulnia et al., 2011; Wang et al., 2013). Tan et al. (2012) suggest the existence of changes in mitochondrial membrane proteomes in response to cold and chemical stresses for normal mitochondrial function. Compared with WT rice, salt tolerance was significantly enhanced in *SaVHAc1* (encode c1 subunit of vacuole H^+^-ATPase)-expressing rice (Baisakh et al., 2012). Some reports show that the ER membrane protein bZIP is released into the Golgi and is then degraded when plants suffer stress; subsequently, degradation products move into cell nuclei and eventually lead to the up-regulation of stress proteins encoded by nuclear genes (Liu and Howell, 2010; Zhang and Wang, 2012; Howell, 2013). Nowadays, researchers increasingly focus on the study of membrane proteins in plants, including phosphoproteomics (Nuhse et al., 2007), regulation of related metabolism in C4 plant (Manandhar-Shrestha et al., 2013), response to stress (Nouri and Komatsu, 2010; Hopff et al., 2013) and signaling (Nie et al., 2015); meanwhile, the technology of mass spectrometry offers convenience in the identification and quantification of membrane proteins.

Wheat (*Triticum aestivum* L.) is a valuable agricultural crop and an important food source for humans. A continual increase in wheat consumption has led to a demand for greater wheat yields to guarantee world food security (Curtis and Halford, 2014). The utilization of heterosis in wheat is still limited due to its complex hereditary basis and self-pollination; however, the chemical hybridizing agent SQ-1 (CHA-SQ-1) offers a new approach to the application of heterosis. Normal fertile male wheat can be made sterile after the spraying of appropriate doses of CHA-SQ-1 at a specific developmental period. The proportion of male sterile wheat can reach 95–100%, and the outcrossing rate can exceed 85%. However, how does CHA-SQ-1 cause male sterility in wheat? In previous studies, attention has been focused more on the anther and pollen in CHA-SQ-1-induced male sterility (Ba et al., 2013, 2014a,b; Wang et al., 2015; Zhu et al., 2015a). Despite some progress in reactive oxygen metabolism (Ba et al., 2013), aliphatic metabolism (Ba et al., 2014a), DNA methylation (Ba et al., 2014b), cell morphology (Wang et al., 2015), and transcriptome (Zhu et al., 2015a), there is a dearth of information on the interceptive mechanism of CHA-SQ-1-induced male sterility. More importantly, the involvement of leaves as the recipient tissues has not yet been reported.

Flag leaves provide sufficient energy and sucrose for meiosis to take place in the pollen mother cells and to allow pollen development and the accumulation of pollen starch during the reproductive stage (Liu et al., 2006; Zhang et al., 2010). SQ-1 is a pyridazine chemical hybridizing agent which can induce male sterility in wheat. During the induction of wheat male sterility, the flag leaves are the initial receptor of CHA-SQ-1, and are stressed by its application. However, after spraying CHA-SQ-1 on flag leaves, the dynamic characteristics of the membrane proteins of the flag leaves remain unknown and the relationship between the dynamic characteristics of membrane proteins and cellular metabolic processes also remain unclear. In order to explore how the membrane proteomes responses to this abiotic stress during CHA-SQ-1-induced male sterility, we profiled the dynamic characteristics of proteomes for flag leaf membrane-enriched fraction in the presence and absence of CHA-SQ-1.

## Materials and Methods

### Plant Materials

The wheat cultivar cv. Xinong 1376 was grown in an experimental field of the Northwest Agriculture and Forestry University, Yangling, China (108° E, 34° 15′ N). The wheat flag leaves were treated with CHA-SQ-1 (5 kg/hectare) when wheat reached the Feeks’ 8.5 stage (the internal morphology of wheat development where the connectivum is formed in female and male primordia). Wheat flag leaves treated with water were used as a control. Flag leaves were collected from control and treated with CHA-SQ-1 (at 2, 4, 6, 10, and 24 h after treatment, respectively) and analyzed for physiological indices. For proteomics, flag leaves treated with CHA-SQ-1 after 2 and 6 h were collected, respectively. Three independent experiments were performed as biological replicates for all experiments.

### Determination of O_2_^-^ Formation Rate and H_2_O_2_ Content

Determination of O_2_^-^, and H_2_O_2_ content were performed according to Ba et al. (2013). Estimation of Superoxide anion, flag leaves (0.5 g) were homogenized in 65 mM phosphate buffer (pH 7.8) and centrifuged at 4°C, 5000 × *g* for 10 min. The supernatants were incubated in 65 mM phosphate buffer (pH 7.8) and 10 mM hydroxylamine chlorhydrate at 25°C for 20 min, and then added 17 mM sulfanilamide and 7 mM α-naphthylamine for another 20 min. The absorbance at 530 nm was measured with a Nicolet Evolution 300 spectrophotometer (Thermo, USA) and the formation rate of O_2_^-^ was calculated from a standard curve of NaNO_2_.

For determination of H_2_O_2_, flag leaves (0.5 g) were homogenized in cold (-20°C) acetone and centrifuged at 4°C, 3000 × *g* for 10 min. The supernatant was added to ammonia and 95% (v/v) hydrochloric acid containing 20% (v/v) titanium tetrachloride. After centrifugation at 10 000 × *g* for 10 min, the sediment was repeatedly washed with cold (-20°C) acetone and centrifuged at 14 000 × *g* for 4°C and then dissolved in 1 M H_2_SO_4_. The absorbance was measured at 410 nm and the content of H_2_O_2_ in the leaves was calculated with an H_2_O_2_ solution-derived standard curve.

### Determination of Activities of Antioxidant Enzymes

The activities of SOD, CAT, and POD were performed according to Ba et al. (2013). To extract antioxidant enzymes, 0.5 g fresh flag leaves were ground in 50 mM cool phosphate buffer [containing 1% (w/v) polyvinylpyrrolidone, pH 7.0] and centrifuged at 4°C, 15 000 × *g* for 20 min. The supernatant was used for assays of enzyme activity.

The activity of SOD was determined by measuring its ability to inhibit the photoreduction of nitro blue tetrazolium (NBT). The reaction solution contained 50 μM NBT, 1.3 μM riboflavin, 13 mM methionine, 75 nM EDTA, 50 mM phosphate buffer (pH 7.8), and enzyme extract. The photo-induced reaction was performed under a light bank at 78 μM m^-2^ s^-1^ for 15 min. The absorbance of the irradiated and non-irradiated solution at 560 nm was determined with a Nicolet Evolution 300 spectrophotometer (Thermo, USA). One unit of SOD activity was defined as the amount of enzyme that would inhibit 50% of NBT photo reduction.

The CAT reaction solution contained 50 mM phosphate buffer (pH 7.0), 15 mM H_2_O_2_, and enzyme extract. Reaction was initiated by adding enzyme extract. Changes in absorbance of the reaction solution at 240 nm were read every 20 s. One unit CAT activity was defined as an absorbance change of 0.01 units per min.

The POD reaction solution contained 50 mM sodium acetate buffer (pH 5.0), 20 mM guaiacol, 40 mM H_2_O_2_, and enzyme extract. Changes in absorbance of the reaction solution at 470 nm were determined every 20 s. One unit POD activity was defined as an absorbance change of 0.01 units per min.

### Determination of MDA Content

The level of lipid peroxidation in samples was determined by estimating the MDA content according to Ba et al. (2013). 0.5 g leaf sample was homogenized in 20% (v/v) trichloroacetic acid and 0.5% (v/v) thiobarbituric acid, and centrifuged at 10 000 × *g* for 10 min. The amount of MDA in the supernatant was estimated by the thiobarbituric acid reaction.

### Measurement of Photosynthesis

Net photosynthetic rates (Pn) of wheat flag leaves were measured with a portable photosynthesis measurement system (Li-6400, Li-Cor, USA) at a photosynthetic photo flux density (PPDF) of 1200 μmol m^-2^ s^-1^ (provided by a red–blue LED light source) and an ambient CO_2_ concentration of 400 μmol mol^-1^.

### Measurement of Total Soluble Sugars and Starch Content

Flag leaves were ground in liquid nitrogen and homogenized with 80% ethanol. After centrifugation at 16 000 × *g* for 10 min, the supernatant was removed to a fresh tube and the pellet was extracted another two times. Starch in the pellet was hydrolyzed with 30% perchloric acid overnight at room temperature, then incubated at 60°C for 10 min and centrifuged at 16 000 × *g* for 10 min. Total soluble sugar and starch content were determined using the anthrone method as described by Zhu et al. (2015b).

### Isolation of Membrane-Enriched Fraction

The membrane fraction was isolated as previously described (Martinec et al., 2000) with few modifications. Approximately, 50 g of tissue was chopped and ground in a Waring blender. The tissue was homogenized in 500 ml homogenizing solution [500 mM sucrose, 50 mM Tris-MES, 50 mM EDTA-Na_2_, 20 mM NaF, 10% glycerol (v/v), 10 mM ascorbic acid, 0.6% PVP (w/v), 1 mM PMSF, 0.5% BSA (w/v), 1 mM DTT; pH 7.8]. The homogenates were filtered through four layers of cheese-cloth and centrifuged at 6000 × *g* for 15 min. Membrane vesicles were pelleted from the resulting supernatant by centrifugation at 150 000 × *g* for 45 min. The resulting pellet containing the membrane fraction was suspended in suspension buffer (50 mM PBS, 330 mM sucrose, 10 mM NaF, and 2 mM DTT; pH 7.8).

### ATPase Activity Measurement

Enrichment assessment of various subcellular membranous components was performed by measurement of membrane-specific H^+^-ATPase activity compared to total ATPase activity. The hydrolytic activity of H^+^-ATPase was determined according to the procedures of Yan et al. (2002) and Shen et al. (2006). Hydrolytic ATPase activity was determined in 0.5 ml Tris/MES buffer containing 50 mM Tris-MES, 1 mM Na_2_MoO_4_, 5 mM MgSO_4_, 50 mM KCl, 0.02% Brij 58 (w/v), 5 mM ATP-Na_2_, pH 6.5 for P-ATPase activity or 8.0 for V-ATPase activity, and F-ATPase activities. Subsequently 1 mM NaN_3_, 50 mM KNO_3_, and 0.5 mM Na_3_VO_4_ were contained respectively for determination of F-ATPase activity, V-ATPase activity, and P-ATPase activity. The reaction was initiated by the addition of the membrane-enriched fraction (equivalent to 3 μg of membrane protein), proceeded for 30 min at 30°C, and stopped with 0.5 ml of stopping reagent (10% SDS). After 2 min, color reagent containing 2% concentrated H_2_SO_4_ (v/v) and 0.5% (NH_4_)_2_MoO_4_ (w/v) was added, followed immediately by 1 ml Millipore-Q water and 10 μl of 10% (w/v) ascorbic acid. Color development was completed after 30 min. To select the most suitable wavelength for the activity of H^+^-ATPase, we employed the full wavelength range for the first optimization using a UV-spectrophotometer. Then, ATPase activity was calculated as phosphate liberated in excess of the boiled membrane fraction control.

### 2-DE and Gel Image Analysis

2D-PAGE was performed following the method of Ye et al. (2013) with minor modifications. The fractions’ membrane proteins were solubilized in lysis solution containing 7 M urea, 2 M thiourea, 4% (w/v) CHAPS, 65 mM DTT, 0.5% (v/v) Bio-Lyte (pH 4–7), and 0.001% (w/v) bromphenol blue. The protein concentration was determined by using a standard curve (*R*^2^ = 0.998) of Bio-Rad Protein Assay Kit II (Bio-Rad, USA), according to the manufacturer’s instructions. About 520 μg of membrane protein was loaded onto a commercially available precast IPG strip (Bio-Rad, USA) with a 17 cm linear pH 4–7 gradient and actively rehydrated at 50 V for 12 h at 20°C. Subsequently, focusing was performed on the IPGphor apparatus (PROTEAN IEF Cell; Bio-Rad, USA) under the following conditions: 250 V for 1 h, 500 V for 1 h, 1000 V for 1 h, 400 V for 1 h, 8000 V for 4 h, and 8000 V to achieve 80000 V-h. Prior to SDS-PAGE, the strips were equilibrated for 15 min in 10 ml of reducing equilibration buffer [6 M urea, 2% SDS (w/v), 0.375 M Tris-HCl at pH 8.8, 20% glycerol (v/v), 2% DTT (w/v)] and then for another 15 min in alkylating equilibration buffer containing 2.5% (w/v) iodoacetamide instead of 2% DTT. The strips were placed on the top of vertical 11% SDS-PAGE. Electrophoresis was carried out at 15°C and 10 mA for 1 h and then at 20 mA until the dye front reached the bottom of the gel. The gels were then stained with Coomassie Brilliant Blue (CBB) G250. Each sample was run in three independent experiments (biological replicates).

Gels were visualized using a PowerLook 2100XL scanner (UMAX, Taiwan, China) at a resolution of 600 dpi. Images were analyzed using the analytical software PDQuest 2-DE 8.0.1 (Bio-Rad, USA) for spot detection, gel matching, and statistical analysis of spots. The selection of protein spots of interest for analysis by MS was based on a fold change ≥1.5 (*p* < 0.05).

### In-Gel Digestion and MALDI-TOF/TOF MS Analysis

The protein spots were excised manually, washed twice with Millipore-Q water, and destained with fresh solution (50% ACN, 40 mM NH_4_HCO_3_). The reaction was stopped with Millipore-Q water when the blue color disappeared after about 2 min. Next, 200 μl of 40 mM ammonium bicarbonate (NH_4_HCO_3_) was added to cover the gel for 2 min, followed by repeated dehydration with changes of 150 μl 100% acetonitrile (ACN) until the gel pieces turned opaque white; the gel was then dried in a vacuum centrifuge. Enzymatic digestion was performed with 10 μg/ml trypsin (enzyme/sample ratio 1:20) during incubation for 45 min in an ice bath, and the supernatant was subsequently removed. Then, 40 μl 40 mM of NH_4_HCO_3_ solution containing 10% ACN was added to cover the gel, followed by incubation for 16 h at 37°C. Following enzymatic digestion, the supernatant was collected and the resultant peptides were extracted during 20 min incubation in 20 μl extraction solution containing 0.1% trifluoroacetic acid (TFA) and 60% ACN. The supernatant was then collected and the peptides were re-dissolved in 20 μl extraction solution (50% ACN, 0.1% TFA) for 10 min. Subsequently, the supernatant was collected and mixed with the previous two supernatants. Mass spectra were collected using a 5800 MALDI Time of Flight (TOF)/TOF^TM^ analyzer with α-cyano-4-hydroxycinnamic acid (CHCA) as matrix and analyzed using TOF/TOF^TM^ Series Explorer^TM^ Software V4.1.0 (AB Sciex, Foster City, CA, USA).

Searches for MS/MS mass spectra were performed using the Mascot search engine^1^ against the NCBInr and Swissprot databases with a taxonomy parameter set to green plants. For the database search, parameters such as one missed cleavage site by trypsin, peptide tolerance of 100 ppm, and an MS/MS tolerance of 0.3 Da, peptide charge of 1+, carbamidomethylation of cysteine, and oxidation of methionine as fixed and variable modifications were used. The MASCOT score, the number of peptide matches, sequence coverage, pI, and molecular weight were used to evaluate the database search results. Sequences of proteins identified as unknown, hypothetical or proteins with an uncharacterized function were used as queries for searching their homologues with BLASTP algorithm.

### Bioinformatic Analysis

The prediction of transmembrane domains (TMDs) of the identified proteins was carried out using TMpred^2^. Grand Average of Hydropathicity (GRAVY) value for each protein was calculated using the Protein GRAVY tool^3^. Hierarchical cluster analysis and heat-map were performed using MultipleExperiment Viewer 4.9 software based on the Log 2-transformed fold change. All identified proteins were blasted against the *Arabidopsis thaliana* TAIR10 (The *Arabidopsis* Information Resource) protein database^4^ with the intention of obtaining annotated protein entries for protein–protein interaction network (PPI) tools. Results with the highest score and lowest E value were considered as relevant for each identified protein. A PPI was constructed with the online analysis tool STRING 9.1 and biological processes and molecular functions were predicted by BiNGO 3.0.2, a plugin for Cytoscape. Subcellular localization of the identified proteins was analyzed using AT_CHLORO database^5^, TAIR10 databas^4^ and SUBA3 database^6^.

### Statistical Analysis

The results presented are the means of three independent experiments. Sample variability is given as the standard deviation of the mean. The significance of differences between control and treatment mean values was determined by Student’s *t*-test, at the 0.01 significance level, and where applicable at the 0.05 significance level.

## Results

### Physiological Changes in Wheat Flag Leaves Under CHA-SQ-1 Treatment Conditions

**FIGURE 1:**
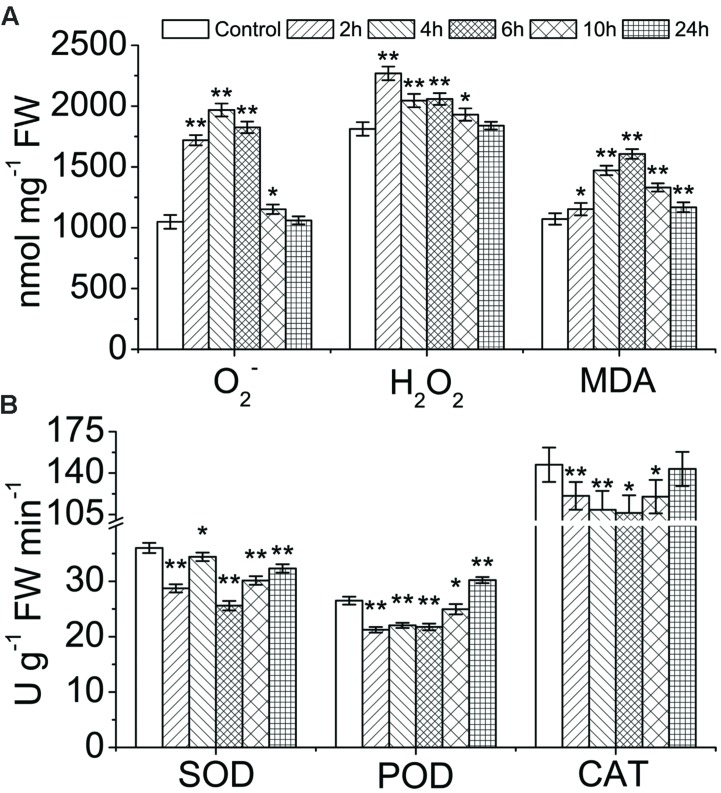
**Assessment of ROS content and oxidative stress analysis in wheat flag leaves under chemical hybridizing **agent (CHA)-**SQ-1 treatment conditions. (A)** The generation rate of O_2_^-^ and content of H_2_O_2_ in control wheat flag leaves and CHA-SQ-1 treated flag leaves; content of malonaldehyde (MDA) in control and CHA-SQ-1 treated flag leaves. **(B)** Activities of superoxide dismutase (SOD), guaiacol peroxidase (POD) and catalase (CAT) in control and CHA-SQ-1 treated flag leaves. Data are means ± SD of three independent experiments (biological replicates). The significant of differences was assessed by Student’s *t*-test (*^∗^P* < 0.05, *^∗∗^P* < 0.01).

O_2_^-^ and H_2_O_2_, as the major members of the ROS family, play vital roles in oxidative damage to plants. In order to further explore whether the generation and accumulation of ROS caused oxidative damage in CHA-SQ-1 treated wheat flag leaves, ROS content and antioxidant enzyme activities were measured (**Figure 1**). CHA-SQ-1-treated flag leaves had significantly higher levels of O_2_^-^, the precursor of most ROSs, than was found in control leaves from 2 to 10 h and contents increased by 1.64-fold at 2 h, 1.88-fold at 4 h, 1.74-fold at 6 h, and 1.09-fold at 10 h, respectively (**Figure 1A**), thus first showing an increase and then a decrease with treatment time and eventually returning to normal levels. Meanwhile, excess O_2_^-^ was catalyzed to form H_2_O_2_. The levels of H_2_O_2_ in leaves treated at 2, 4, 6, and 10 h after CHA-SQ-1 were also increased by 1.25-fold, 1.12-fold, 1.14-fold, and 1.07-fold, respectively, as compared to the control plants (**Figure 1A**). Meanwhile, the activities of SOD, POD, and CAT were measured, and the results showed that activities of all three antioxidant enzymes in wheat flag leaves declined significantly after CHA-SQ-1 treatment when excess ROS was generated (**Figure 1B**) and this further interfered with the oxidative/antioxidative balance. More importantly, excessive ROS can oxidize membrane lipids and generate MDA, which can aggravate damage to membrane structure. Not surprisingly, within 24 h after CHA-SQ-1 treatments, the levels of MDA were increased by 1.25-fold at 2 h, 1.13-fold at 4 h, 1.13-fold at 6 h, 1.07-fold at 10 h, and 1.09-fold at 24 h over control leaves, respectively (**Figure 1A**).

To determine the effects of CHA-SQ-1 treatment on photosynthesis of flag leaves, net photosynthesis rate (Pn) was measured at different times after CHA-SQ-1 treatment. Meanwhile, contents of soluble sugar and starch were also determined. Results showed that the Pn of the wheat flag leaves decreased significantly from 20.57 μmol m^-2^ s^-1^ (control plants) to 3.11 (at 10 h after CHA-SQ-1 treatment) and began to increased slightly at 24 h after treatment (Supplementary Figure S1). Contents of soluble sugar and starch were decreased by 1.73- to 4.19-fold and 3.26- to 8.01-fold, respectively (Supplementary Figure S2).

### Enrichment Assessment of Various Subcellular Membranous Components

**FIGURE 2:**
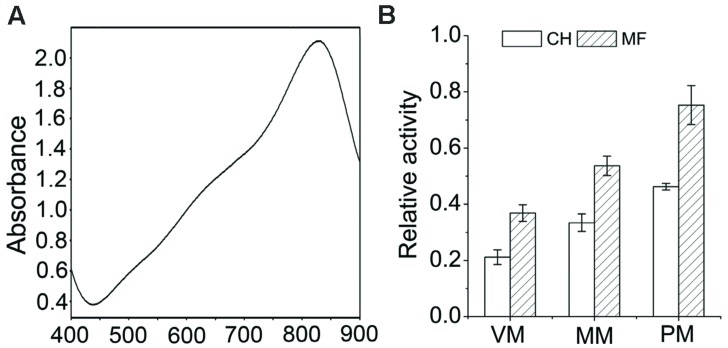
**Enrichment assessment of various subcellular membranous components. (A)** Wavelength scanning of reaction system of H^+^-ATPase activity determination at a wavelength of 400–900 nm. **(B)** Enzymatic characterization of the membrane fraction. Relative H^+^-ATPase activity was determined by measurement of membrane-specific H^+^-ATPase activity compared to total ATPase activity. MF, membrane fraction; CH, crude homogenate; VM, vacuole membrane; PM, plasma membrane; MM, mitochondria membrane. Data are means ± SD of three independent experiments (biological replicates).

Enrichment of the membrane microsomal fraction of flag leaves was accomplished by differential centrifugation. It was systematically assessed for the enrichment of various subcellular membranous components using standard marker enzyme assays viz., vanadate-, azide-, nitrate-sensitive ATPase PM, mitochondrial membrane, tonoplast, and Golgi membranes, respectively. The results showed that a maximum absorption at 820 nm (**Figure 2A**), which means 820 nm was selected as the most adequate wavelength for testing all activity of H^+^-ATPase. Accordingly, the relative changes in percent inhibition of ATPase activities associated with mitochondria, tonoplast, and PM were 1.60-, 1.63-, and 1.73-fold, respectively in microsomal fractions compared to the crude homogenate (**Figure 2B**). The higher level activities of the marker enzymes in the membrane fraction indicate the enrichment of various subcellular membranous components.

### Analysis of Differential Membrane Proteins in Control and CHA-SQ-1 Treated Wheat Flag Leaves

To understand the membrane proteome response to short-term CHA-SQ-1-treatment of wheat flag leaves, and the changes in membrane proteomes of wheat flag leaves from the control, CHA-SQ-1-treated plants (2 and 6 h) were analyzed by 2-DE. The membrane protein maps produced from three independent protein extractions showed a high reproducibility based on analysis using PDQuest software.

**FIGURE 3:**
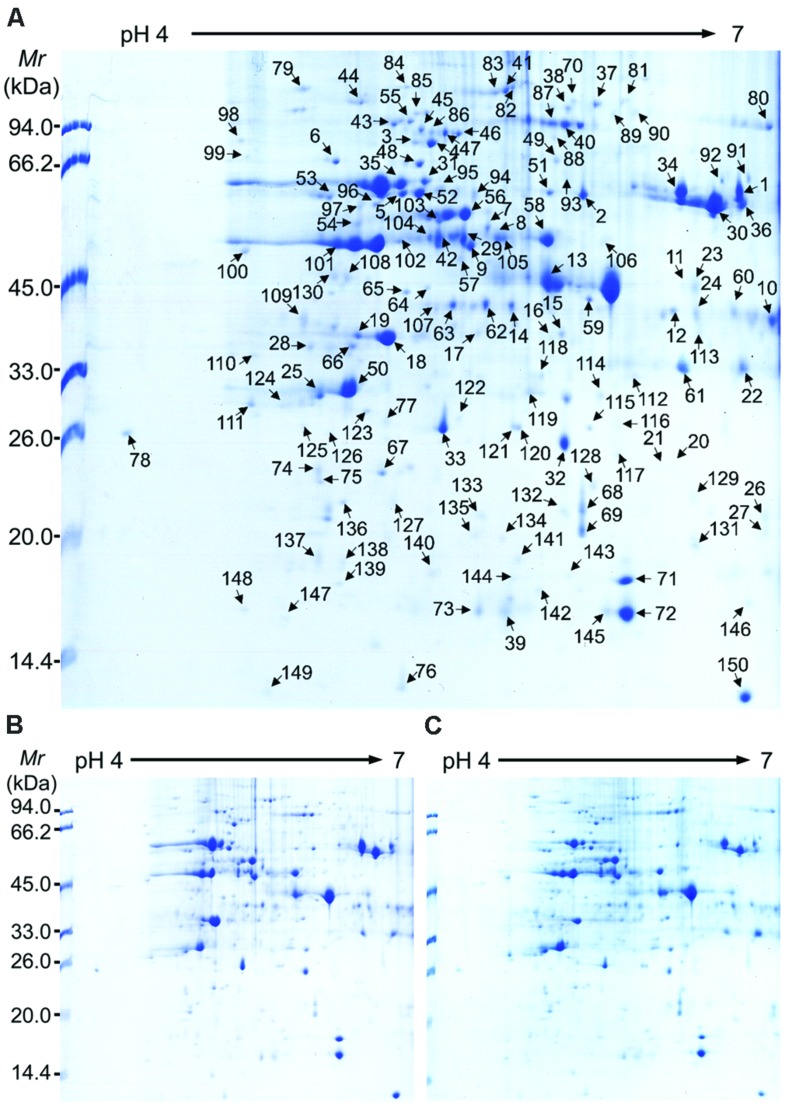
**2-DE image analysis of membrane proteomes in control and CHA-SQ-1-treated wheat flag leaves. (A)** 2-DE map of control flag leaves; **(B)** 2-DE map of flag leaves treated with CHA-SQ-1 for 2 h **(C)** 2-DE map of flag leaves treated with CHA-SQ-1 for 6 h.

**Figure 3** shows a representative gel image of proteins extracted from the control and CHA-SQ-1-treated plants. Protein spots [345 (±12), 361 (±15), 370 (±13)] were reproducibly detected using PDQuest software from the control, 2 and 6 h after CHA-SQ-1 treatment, respectively (*n* = 3). From a spot-to-spot comparison and based on statistical analysis, a total of 150 spots (numbered from 1 to 150) exhibited at least 1.5-fold (*p* < 0.05) difference in abundance between the control and CHA-SQ-1 treatment (**Figure 3**, Supplementary Table S1). In wheat leaves at 2 h after treatment, 68 spots had >1.5-fold change in abundance (*p* < 0.05) and 37 spots showed a >2.0-fold change; meanwhile, in wheat leaves at 6 h after CHA-SQ-1 treatment, 83 spots had a >1.5-fold change in abundance and 41 spots showed a >2.0-fold change (Supplementary Table S1). Among 150 differential proteins, only spot 20 showed qualitative changes and was detected in CHA-SQ-1-treated flag leaves (**Figure 3**, Supplementary Table S1).

**FIGURE 4:**
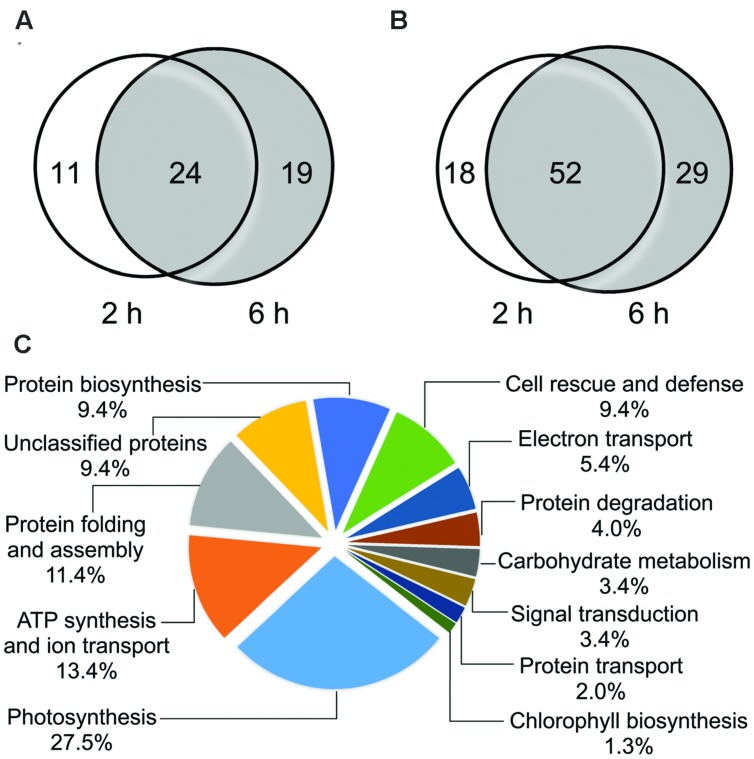
**Venn diagram analysis of the differentially expressed membrane protein spots and functional classification of 149 identified membrane proteins. (A)** The numbers of differentially expressed protein spots with down-regulation in wheat flag leaves after CHA-SQ-1 treatment for 2 and 6 h. **(B)** The numbers of differentially expressed protein spots with up-regulation in wheat flag leaves after CHA-SQ-1 treatments for 2 and 6 h. **(C)** Distribution of proteins according to their biological functions. Unclassified proteins include those whose functions have not been described.

To understand the differentially expressed protein (DEP) profile patterns at two time points during CHA-SQ-1 treatment, the distribution of proteins were analyzed. **Figures 4A,B** present the number of DEPs under CHA-SQ-1 treatments and how these spots overlap using Venn diagram analysis. There were 35 up-regulated proteins and 70 down-regulated proteins in flag leaves at 2 h after CHA-SQ-1 treatment; meanwhile, there were 43 up-regulated proteins and 81 down-regulated proteins in flag leaves at 6 h after CHA-SQ-1 treatment (**Figures 4A,B**). Among the up-regulated spots, 24 spots were up-regulated at both CHA-SQ-1 time treatments; of all down-regulated spots, 52 spots were down-regulated under both two different CHA-SQ-1 treatments (**Figures 4A,B**). Approximately 50% (52.5% for down-regulated proteins and 44.4% for up-regulated proteins) of the DEPs exhibited a similar regulatory pattern under the two different CHA-SQ-1 treatments. Additionally, 77 spots showed up or down-regulation under only one treatment. Of these, 29 spots were found to be differentially expressed at 2 h after CHA-SQ-1 treatment, while the other 48 spots showed significant changes in response to 6 h of CHA-SQ-1 treatment, indicating that these spots were specifically responsive to short-term CHA-SQ-1-treatment of wheat flag leaves (**Figures 4A,B**).

### Identification and Functional Classification of DEPs

A total of 150 DEPs were analyzed by MALDI-TOF/TOF MS. Amongst them, 149 were successfully identified by MS/MS (Supplementary Table S2). Among the 149 identities, 142 have been functionally annotated in the current database, whereas the remaining seven identities were either unnamed proteins (spot 84, spot 92, spot 115, and spot 120) or hypothetical proteins (spot 27, spot 78, and spot 109; Supplementary Table S2). To annotate their identities, their sequences were used as a query to search for homologs using BLASTP (NCBI). The corresponding homologs with the highest similarity are listed in Supplementary Table S3. All 7 proteins shared at least 80% sequence similarity, suggesting that they may have similar function with their homologues. In summary, 149 identities represented 103 unique proteins.

**FIGURE 5:**
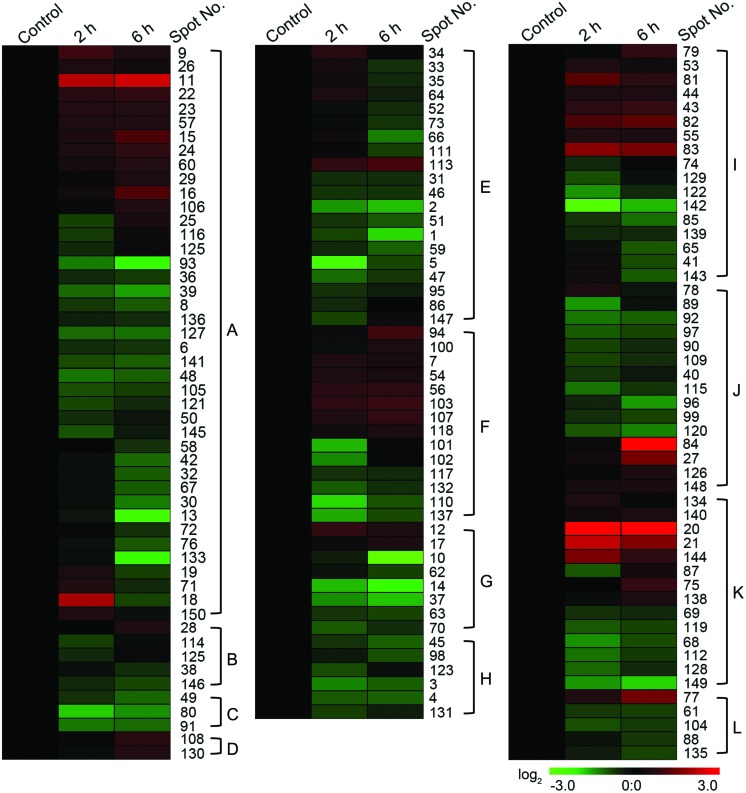
**Hierarchical clustering of membrane proteins of all 12 categories in control and two different CHA-SQ-1 treatment regimes of wheat flag leaves**. The hierarchical cluster analysis was conducted using MultiExperiment Viewer 4.9 and the Log-transformed values of -fold change ratios listed in Supplementary Table S4. A, photosynthesis; B, signal transduction; C, protein transport; D, chlorophyll biosynthesis; E, ATP synthesis and ion transport; F, protein biosynthesis; G, electron transport; H, protein degradation; I, protein folding and assembly; J, unclassified proteins; K, cell rescue and defense; L, carbohydrate metabolism.

Based on the metabolic and functional features of wheat flag leaves, all of the 149 identities were classified into 12 major categories, including photosynthesis, ATP synthesis and ion transport, protein folding and assembly, unclassified proteins, protein biosynthesis, cell rescue and defense, redox homeostasis, carbohydrate metabolism, protein degradation, signal transduction, protein transport, and chlorophyll biosynthesis (**Figure 4C**). Eighty percent of these identified proteins were implicated in the first six functional groups, whereas the largest functional groups that were greatly affected by CHA-SQ-1 treatment were proteins involved in photosynthesis (27.5%). Further analysis of the change of abundance in each group revealed that proteins involved in ATP synthesis (13.4%), protein folding and assembly (11.4%), protein biosynthesis (9.4%), and stress-related proteins (9.4%) were overrepresented, either in number or in expression level, suggesting that these processes were susceptible to CHA-SQ-1 treatment. In order to visualize the protein expression patterns of all 12 categories, hierarchical clustering of proteins was analyzed (**Figure 5**).

In general, the apparent *M*r predicted by SDS-PAGE has an error of about ±10% compared with the theoretical value. However, amongst all of the identified proteins, a set of 37 identities with known function were found with observed *M*r values much smaller than the theoretical values (Supplementary Table S2), suggesting that these proteins appeared to be partially degraded products of their intact proteins. Of these, twelve identities were involved in photosynthesis (spot 26, spot 39, spot 48, spot 67, spot 71, spot 72, spot 76, spot 116, spot 121, spot 133, spot 141, and spot 145); five identities were involved in the process of protein folding and assembly (spot 74, spot 129, spot 139, spot 142, and spot 143); respectively, four identities were involved in the process of ATP synthesis (spot 33, spot 64, spot 66, and spot 111), protein biosynthesis (spot 107, spot 118, spot 132, and spot 137) and cell rescue and defense (spot 68, spot 69, spot 119, and spot 138); three identities were involved in the process of carbohydrate metabolism (spot 61, spot 77, and spot 135); two identities were related to signal transduction (spot 124 and spot 146); and respectively, one identity was suggested to be related to protein degradation (spot 123) and protein transport (spot 49) processes; one identity (spot 27) was unclassified protein.

By contrast, 17 identities with annotated function were found with observed *M*r values much larger than theoretical values (Supplementary Table S2), indicating that these proteins may be products of post translation modified proteins.

### Physicochemical Characteristics of Membrane Proteins

**FIGURE 6:**
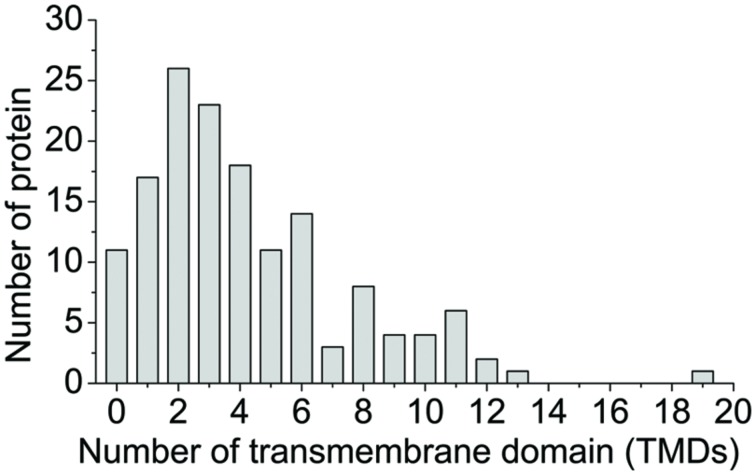
**Distribution of the number of predicted transmembrane domains (TMDs) of membrane protein in wheat leaves**. TMDs analysis of identified proteins was performed using TMpred program. The algorithm is based on the statistical analysis of TMbase. The number of TMDs for every DEP was listed in Supplementary Table S2.

To evaluate the physicochemical characteristics of flag leaf membrane proteins, all of the identified proteins were analyzed in terms of hydrophobicity (GRAVY values) and the number of TMDs. The GRAVY score takes into account the size and the charge of the whole protein and ranges for instance from -2 to +2, positive values referring to hydrophobic proteins while negative values refer to hydrophilic proteins. The majority of analyzed proteins have a GRAVY between -0.4 and +0.4, which could not discriminate their hydrophobic or hydrophilic nature (Morel et al., 2006). In our case, the GRAVY of membrane proteins of the wheat flag leaves analyzed ranged from -1.122 to +1.125, and most of the proteins (78.5%) had a GRAVY index between -0.4 and +0.4 (Supplementary Table S2). TMDs prediction programs (TMpred) were used to predict putative TMDs in all of 149 identified proteins (Supplementary Table S2). TM candidate proteins formed 92.7% (138/149) of the proteins, displaying at least one TMD. Among them, 47.8% (66/138) exhibited one to three TMDs, 31.2% (43/138) showed four to six TMDs, and 21.0% (29/138) had 7 to 19 TMDs (**Figure 6**). It has been reported that most of the integral cytoplasmic membrane proteins are hydrophobic, while the majority of integral outer membrane proteins are hydrophilic (Nouwens et al., 2000; Santoni et al., 2000), which can cause the observed ambiguity between GRAVY values and TMDs. A similar phenomena was also observed in our study. Eighty-seven percent (120/138) of proteins with putative TMDs showed negative GRAVY values; while LRR disease resistance protein/transmembrane receptor kinase PS4 with no predicted TMD scored positive GRAVY values (1.125; Supplementary Table S2). According to the localization tools, the results indicated that identified proteins were higher enrichment in membrane proteins, but a number of non-membrane proteins were identified in this study (Supplementary Table S5), which is a common problem in isolating membrane from plant tissue and also observed in many other membrane proteomic studies (Li et al., 2012; Manandhar-Shrestha et al., 2013; Nie et al., 2015).

### PPI Analysis of Identified Membrane Proteins

In order to explore the relationship among all identified differential proteins, protein–protein interaction was analyzed. All 149 identified proteins were blasted against the *Arabidopsis thaliana* TAIR10 protein database (Supplementary Table S5). Identified proteins were grouped into functional classes according to the biological processes in which they are involved. STRING and BiNGO were used to visualize the protein–protein interaction, biological pathways, and molecular functions (Maere et al., 2005; Franceschini et al., 2013).

**FIGURE 7:**
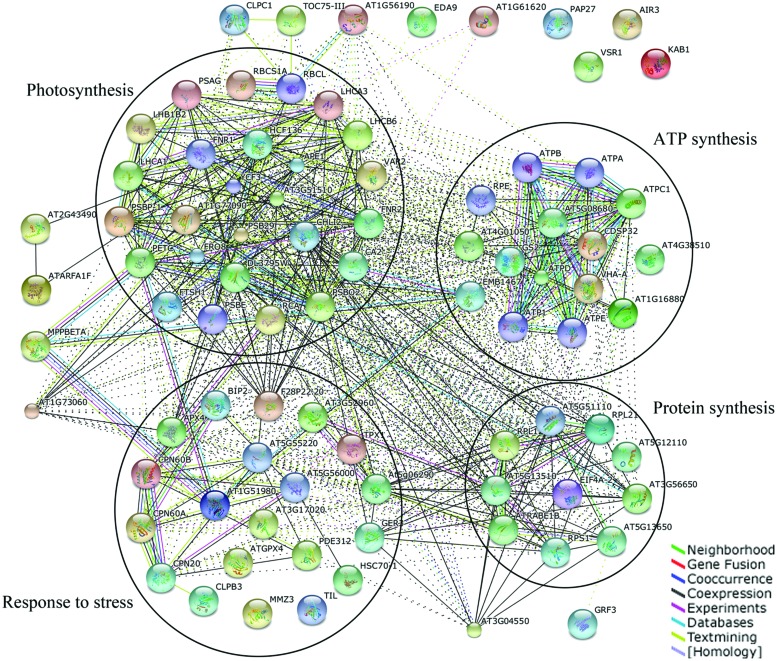
**Analysis of protein interaction network by STRING 9.1**. TAIR homologous proteins from identified proteins were mapped by searching the STRING 9.1 software with a confidence level of 0.4. Colored lines between the proteins indicate the various types of interaction evidence.

The STRING analysis revealed the functional links between different proteins in which proteins involved in photosynthesis, ATP synthesis, response to stress, and protein synthesis were major clusters (**Figure 7**). Actually, these four clusters were not separated and together they formed a related-network in response to CHA-SQ-1 treatment. Proteins overlapped among the four clusters, especially proteins involved in photosynthesis and energy metabolism. Abbreviations of the specific protein names in the network are shown in Supplementary Table S6.

**FIGURE 8:**
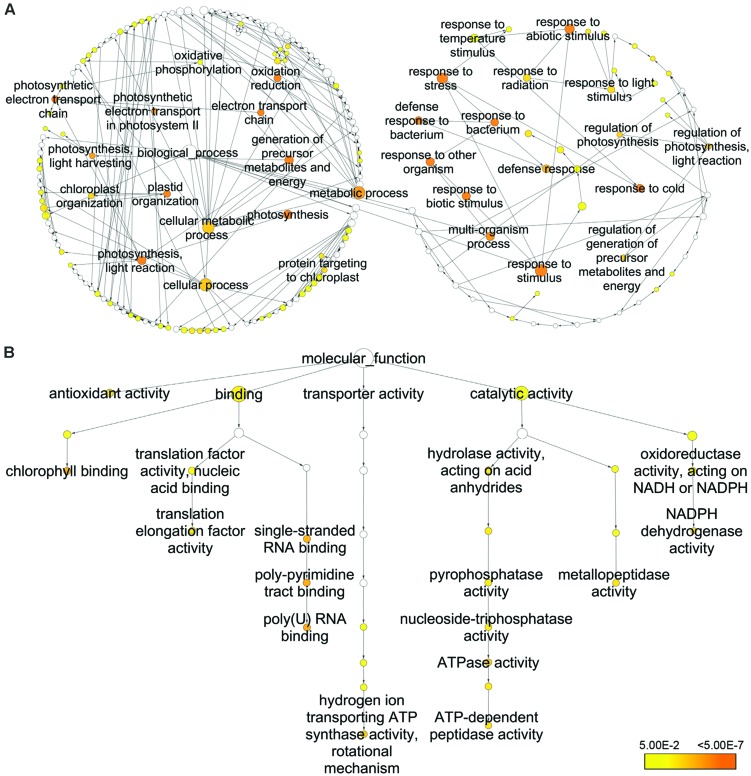
**Biological pathway **(A)** and molecular function **(B)** networks generated by BiNGO**. Homologous proteins were used for the GO analysis. The size of the node is related to the number of proteins and the color represents the p-value for the statistical significance of the overrepresented GO term (see the color scale on the right bottom), whereas white nodes are not enriched.

To obtain statistically over- or under-represented categories of biological pathways and molecular functions related to CHA-SQ-1 treatment, BiNGO was used to analyze identified differential proteins (**Figure 8**, Supplementary Tables S7 and S8). Several overrepresented biological pathways were mostly significant (**Figure 8A**, Supplementary Table S7), including photosynthesis (*p* = 2.3849e–21), photosynthesis and light reaction (*p* = 2.8509e–21), generation of precursor metabolites and energy (*p* = 5.2254e–16), and response to stimulus (*p* = 6.6954e–13). Meanwhile, a complete list of the enriched Gene Ontology (GO) molecular functions for the proteins was presented in **Figure 8B** and Supplementary Table S8. Of them, several most highly enriched molecular functions are poly(U) RNA binding (*p* = 1.5997e–7), poly-pyrimidine tract binding (*p* = 1.5997e–7), chlorophyll binding (*p* = 5.8727e–7), single-stranded RNA binding (*p* = 8.3523e–7), hydrogen ion transporting ATP synthase activity and rotational mechanism (*p* = 5.2896e–6), ATPase activity (*p* = 5.4790e–6), NADPH dehydrogenase activity (*p* = 2.5621e–5), antioxidant activity (*p* = 4.1425e–5), ATP-dependent peptidase activity (*p* = 4.1601e–5), metallopeptidase activity (*p* = 6.8133e–5) and translation elongation factor activity (*p* = 7.6145e–5).

## Discussion

### Oxidative Stress of Wheat Flag Leaves Caused by CHA-SQ-1

Various abiotic stresses lead to the overproduction of ROS in plants, which result in oxidative stress and cause damage to multiple cellular components such as proteins (Gill and Tuteja, 2010). It has been reported previously that CHA-SQ-1, in a manner similar to other stress factors, can generates excessive ROS during the anther abortion (Ba et al., 2013). And, indeed, our current results showed that CHA-SQ-1 also induced the overproduction of ROS in flag leaves and further led to degradation of multiple membrane proteins (Supplementary Table S2). Generally, in order to cope with continuous ROS production under stress, major antioxidant enzymes including SOD, POD, and CAT, are activated to scavenge ROS (Gill and Tuteja, 2010). We found an inverse correlation between the level of intracellular ROS and the activities of leaf SOD, CAT, and POD. This meant that excessive ROS cannot be eliminated effectively at the early stage of CHA-SQ-1 treatment, which aggravated damage to cellular components. In plants, overproduction of ROS can destroy membrane stability through the formation of MDA (Gill and Tuteja, 2010). Similarly, in the present study, MDA content increased significantly under CHA-SQ-1 treatment, showing that the peroxidative reaction in membrane lipids had become stronger and the plant injury would be more serious. In this case, membrane proteomes were damaged.

### Proteins Involved in Photosynthesis

Photosynthesis is the primary pathway for the production of carbohydrates, which are essential for cell growth and proliferation. It is composed of two steps, photoreaction (light dependent) and dark reaction (carbon fixation). The capability of a plant to maintain a stable photosynthetic rate is significant for sustaining plant growth under stress conditions (Wang et al., 2013). Previously, our results suggest that CHA-SQ-1 affected the major products of photosynthesis during the anther abortion, such as sucrose, starch and aliphatic metabolism (Ba et al., 2014a; Zhu et al., 2015b). Similarly, in the present study, 41 DEPs were found to be associated with the photosynthetic process under CHA-SQ-1 treatment (Supplementary Table S2).

Of these, six DEPs (spot 25, spot 50, spot 67, spot 121, spot 127, and spot 136) belonged to Chlorophyll a/b binding proteins (LHCB; Supplementary Table S2). As a whole, their abundance was down-regulated in CHA-SQ-1 treated flag leaves (Supplementary Table S1). LHCB are the most abundant membrane proteins in plants and play a vital role in maintaining a stable photosynthetic rate. Previous study showed that gene expression of LHCB is down-regulated under ROS stress (Staneloni et al., 2008), and decrease LHCB expression leads to plants being more vulnerable under stress conditions (Andersson et al., 2001; Ganeteg et al., 2004; Kovács et al., 2006).

Oxygen evolving enhancer proteins 1 (OEE 1) and OEE 2 are responsible for the stability of PS II and play a vital role in catalyzing the splitting of water (Mayfield et al., 1987a,b). Study has shown that OEE 2 expression is increased in some species under drought and salt stress (Sugihara et al., 2000; Abbasi and Komatsu, 2004; Gazanchian et al., 2007). However, a few studies have shown that the expression level of OEE decreased during salt stress in potatoes and wheat (Aghaei et al., 2008; Gao et al., 2011). In our study, the abundance of OEE 1 (spot 18 and spot 19) was up-regulated at 2 h after CHA-SQ-1 treatment, and down-regulated at 6 h after CHA-SQ-1 treatment (Supplementary Tables S1 and S2). At 2 h after CHA-SQ-1 treatment, increased abundance of OEE 1 helped repair the damaged PS II. However, decreased abundance of OEE1 at 6 h after treatment caused instability of PS II under peroxide stress caused by CHA-SQ-1. Meanwhile, the abundance of OEE 2 (spot 32) was reduced at 6 h after CHA-SQ-1 treatment in flag leaves (Supplementary Tables S1 and S2), which further slowed the photosynthetic process.

### Proteins Involved in ATP Production and Electron Transport

ATP synthase is the universal enzyme that manufactures ATP from ADP and provides energy for a large number of fundamental biological processes (Kang et al., 2012). In the present study, a total of 16 DEPs were involved in ATP synthesis, with 14 DEPs down-regulated and two DEPs (spot 34 and spot 64) up-regulated in response to CHA-SQ-1 treatment of flag leaves (**Figure 3**, Supplementary Tables S1 and S2). During biotic and abiotic stresses in plants, high energy costs are required at the stage of stress acclimation, such as the increased relative abundance of components of mitochondrial ATP-synthase (Kosová et al., 2014). Here, as a whole, the abundance of different subunits of ATP synthesis was decreased in CHA-SQ-1 treated flag leaves compared to the control plant; this interrupted multiple normal metabolic processes dependent on ATP. In addition, the abundance of vacuolar proton-ATPase subunit A (spot 46, spot 47, and spot 86) was decreased significantly in CHA-SQ-1 treated flag leaves (**Figure 3**, Supplementary Tables S1 and S2), which would cause an imbalance of homeostasis and abnormal cellular activity. These negative events might trigger an insufficiency of ATP production for maintaining normal metabolic processes under CHA-SQ-1 treatment in flag leaves.

The process of ATP production is always along with electron transport. Ferredoxin-NADP^+^ oxidoreductase (FNR) is a ubiquitous enzyme encoded by nuclear genes in higher plants. It catalyzes reversible electron transfer between ferredoxin (Fd; or flavodoxin) and NAD(P)H (Mulo, 2011). In the present study, the expression level of FNR (spot 10, spot 12, spot 14, spot 62, and spot 63) was decreased in CHA-SQ-1 treated wheat flag leaves compared to the control plants (Supplementary Tables S1 and S2). As mentioned above, LHCB was down-regulated under CHA-SQ-1 treatment, and this decrease may hinder photon capturing and the transfer the excitation energy to reaction centers to decrease NADP^+^ to NADPH generation (Komatsu et al., 2014). Subsequently, the marked release of FNR from the thylakoid membrane followed by a reduction in NADP^+^ photoreduction capacity might maintain the NADP^+^/NADPH homeostasis of the stressed plants (Palatnik et al., 1997; Lehtimäki et al., 2010). On the other hand, decreased reduction of NADP^+^ caused the photosynthetic electron transport chain to become over-reduced, therefore accelerating the formation and accumulation of ROS (Komatsu et al., 2014). As a consequence, the photosynthetic process was further impaired and slowed the growth of wheat plants and the development of pollen. In addition, NADH-ubiquinone oxidoreductase (spot 37) was down-regulated in CHA-SQ-1 treated flag leaves in our study (**Figure 3**, Supplementary Tables S1 and S2). NADH-ubiquinone oxidoreductase is an enzyme that detoxifies quinones and their derivatives (Wang et al., 2013) and its down-regulation in CHA-SQ-1 treated flag leaves might cause further damage to cellular metabolic process.

### Proteins Involved in the Stress Response

In the present study, ROS and MDA, with the potential to cause cellular damage, were generated and accumulated. Under abiotic stresses, plants have evolved protective mechanisms to eliminate or reduce ROS and MDA levels and defend against a stressful environment (Gill and Tuteja, 2010). In our study, some anti-stress proteins, such as Temperature stress-induced lipocalin (TIL; spot 134) and 2-Cys peroxiredoxin (spot 138 and spot 140), and Abscisic acid stress ripening (ASR; spot 20 and spot 21) who play vital roles in response to stresses (König et al., 2002; Yang et al., 2005; Abo-Ogiala et al., 2014), were up-regulated to response to CHA-SQ-1 treatment in flag leaves (**Figure 3**, Supplementary Tables S1 and S2). To some extent, this increase can restrain accumulation of ROS. However, in our results, other antioxidative proteins, such as LRR disease resistance protein (spot 87 and spot 149) were down-regulated in CHA-SQ-1 treated flag leaves (**Figure 3**, Supplementary Tables S1 and S2). This means that excessive ROS was not been completely scavenged and this aggravated the damage to cellular biological processes.

### Protein Metabolism-Related Proteins

Synthesis, assembling, folding, and degradation-related proteins are necessary for maintaining cellular protein homeostasis (Saikawa et al., 2004). In the present study, DEPs involved in protein metabolism are implicated in three functional subgroups: (1) protein synthesis-related proteins; (2) assembling/folding-related proteins; (3) degradation-related proteins.

In the first subgroup, five elongation factor Tu (EF-Tu; spot 7, spot 54, spot 56, spot 103, and spot 118) were up-regulated in flag leaves under CHA-SQ-1 treatment (**Figure 3**, Supplementary Tables S1 and S2). EF-Tu is a protein that plays a central role in the elongation phase of protein synthesis in plants. In additional, EF-Tu can prevent aggregation of denatured proteins caused by environmental stresses and chemicals (Kudlicki et al., 1997; Fu and Dooner, 2002). In our case, on the one hand, the up-regulation of EF-Tu could accumulate synthesis of proteins and replaced damaged proteins under CHA-SQ-1 treatment; on the other hand, it prevented the aggregation of damaged proteins caused by ROS.

In the second subgroup, eight DEPs (spot 43, spot 44, spot 55, spot 79, spot 81, spot 82, spot 83, and spot 122) were identified as heat shock proteins (HSPs) and chaperones and their expression levels were increased except for the 20 kD chaperonin (spot 122) whose abundance was decrease in flag leaves under CHA-SQ-1 treatment (**Figure 3**, Supplementary Tables S1 and S2). HSPs and chaperones play crucial roles in protecting plants against stress and they are involved in a wide range of crucial cellular processes (Wang et al., 2004; Al-Whaibi, 2011). Our results indicated that the accumulation of ROS caused instability of proteins and membrane structure under CHA-SQ-1 treatments. An increased abundance of HSPs was a protective mechanism in response to oxidative stress. However, HCF136 (spot 65) which is related to the stability of PS II, was down-regulated in CHA-SQ-1-treated wheat leaves (**Figure 3**, Supplementary Tables S1 and S2) and this might cause the damage of PS II.

In the third subgroup, four DEPs (spot 3, spot 4, spot 45, and spot 98) identified as cell division protease FtsH-like proteins (Supplementary Table S2). FtsH proteins are involved in photosynthesis and control of the cell cycle in eukaryotic cells. *In vivo*, studies have shown that FtsH proteins are responsible for the degradation of unassembled proteins (Ostersetzer and Adam, 1997) and D1 proteins (Lindahl et al., 2000; Malnoë et al., 2014). In the present study, down-regulation of FtsH protein in CHA-SQ-1 treated wheat flag leaves interrupted the process of photosynthesis and impacted the formation of thylakoid membranes (Supplementary Table S1). Meanwhile, variegation in wheat flag leaves after CHA-SQ-1 treatment may be related to a decrease in the abundance of FtsH protein (Chen et al., 2000). These events resulted in there being a low photosynthesis rate (Supplementary Figure S1) and insufficient source of energy (Supplementary Figure S2) to response to CHA-SQ-1 treatment in wheat flag leaves.

### Other Proteins

In addition to the above described DEPs, 14-3-3 protein (spot 28) related to signal transduction was up-regulated in flag leaves after CHA-SQ-1 treatment (**Figure 3**, Supplementary Tables S1 and S2), which suggested signal transduction was enhanced in response to CHA-SQ-1 treatment; Toc75 (spot 80) is the protein translocation channel located in the outer envelope membrane of plastids (Baldwin et al., 2005) and its abundance was decreased in flag leaves after CHA-SQ-1 treatment (**Figure 3**, Supplementary Tables S1 and S2), which resulted in precursor proteins not being transported in the chloroplast stroma and this subsequently impaired chloroplast function.

## Conclusion

During pollen development, wheat flag leaves are an important component of the source-sink unit as they provide sucrose and energy, and initial recipient tissue of CHA-SQ-1. The results of the present work have shown that ROS (O_2_^-^ and H_2_O_2_) contents accumulated rapidly in wheat flag leaves after CHA-SQ-1 treatment and exceeded the cell threshold. An increase in ROS content was accompanied by an inhibition of SOD, CAT and POD activities. Excessive ROS were not effectively removed by the antioxidative system, which induced oxidative damage in membrane structures and membrane proteins. Comparative membrane proteomic analysis revealed that four biological processes (photosynthesis, ATP production, response to stress, and proteins metabolism) were interrupted after CHA-SQ-1 treatment in flag leaves. These abnormal biological processes caused complete dysfunction of the flag leaves. And then dysfunctional cells of flag leaf affected photosynthesis, energy supply and carbohydrate production. These results provide the basic insight needed to further investigate the mechanism of anther abortion induced by CHA-SQ-1.

## Author Contributions

QS, SW, and GZ conceived the study. QS and SW performed the experiments. SW performed the MS/MS analysis. QS carried out the analysis of the data, made the identification of the proteins and drafted the manuscript. QS and SW contributed in the preparation of the final draft of the manuscript. QS, SW, YL, ZL, JG, SM, JW, and NN provided reagents, materials and analysis tools. All authors read and approved the final manuscript.

## Conflict of Interest Statement

The authors declare that the research was conducted in the absence of any commercial or financial relationships that could be construed as a potential conflict of interest.

## Footnotes

1 www.matrixscience.com

2 http://www.ch.embnet.org/software/TMPRED_form.html

3 http://www.bioinformatics.org/sms2/protein_gravy.html

4 http://www.arabidopsis.org/

5 http://at-chloro.prabi.fr/at_chloro/

6 http://suba3.plantenergy.uwa.edu.au/
