# Supplementary material for: Comparative proteomic analysis of a membrane-enriched fraction from flag leaves reveals responses to chemical hybridization agent SQ-1 in wheat
Source: Front Plant Sci. 2015 Aug 26;6:669. doi: 10.3389/fpls.2015.00669 (PMC4549638; doi:10.3389/fpls.2015.00669)
Supplement: Supplementary file 10 [file Image_1.PDF]

Supplementary Material - Figures

Comparative proteomic analysis of a membrane-enriched fraction from flag leaves reveals responses to chemical hybridization agent SQ-1 in wheat

Qilu Song†, Shuping Wang†, Gaisheng Zhang*, Ying Li, Zheng Li, Jialin Guo, Na Niu, Junwei Wang and Shoucai Ma

College of Agronomy, Northwest A&F University, National Yangling Agricultural Biotechnology & Breeding Center, Yangling Branch of State Wheat Improvement Centre, Wheat Breeding Engineering Research Center, Ministry of Education, Key Laboratory of Crop Heterosis of Shaanxi Province, Yangling, Shaanxi 712100, P.R. China

*Correspondence: Gaisheng Zhang, College of Agronomy, Northwest Agriculture and Forestry University, National Yangling Agricultural Biotechnology & Breeding Center, Yangling 712100, P.R. China.

zhanggaisheng18@sohu.com,

† These authors have contributed equally to this work.

Supplementary Figure S1

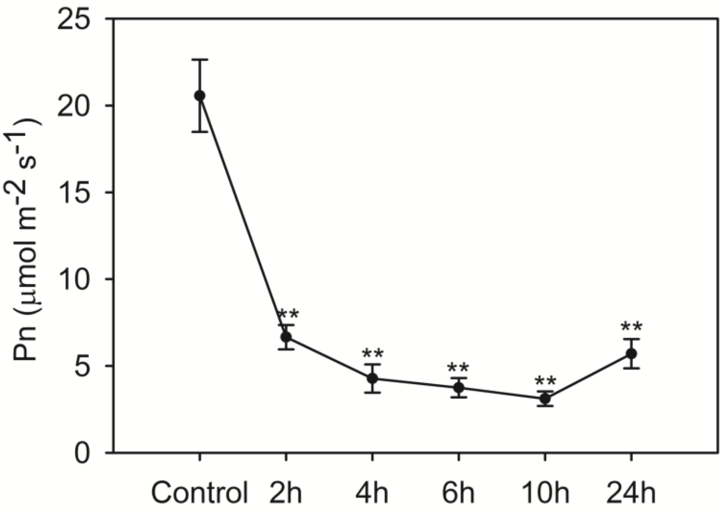

Supplementary Figure S1. Net photosynthesis rate (Pn) in control and CHA-SQ-1 treated flag leaves. Control, 2 h, 4 h, 6 h, 10 h, and 24 h represent the time after CHA-SQ-1 treatment. Data are means ± SD of three independent biological replicates. The significant of differences between control and CHA-SQ-1-treated plants was assessed by Student's t test (*$P<0.05$, **$P<0.01$).

Supplementary Figure S2

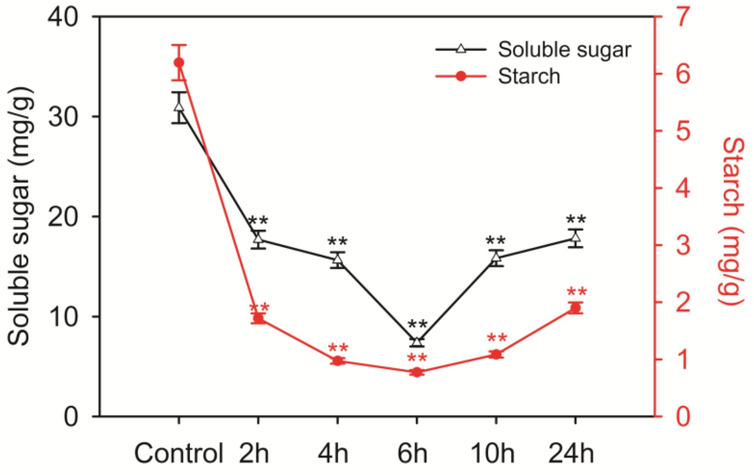

Supplementary Figure S2. Contents of soluble sugar and starch in control and CHA-SQ-1 treated flag leaves. Control, 2 h, 4 h, 6 h, 10 h and 24 h represent the time after CHA-SQ-1 treatment. Data are means ± SD of three independent biological replicates. The significant of differences between control and treatment plants was assessed by Student's t test (*$P<0.05$, **$P<0.01$).

Supplementary Figure S3

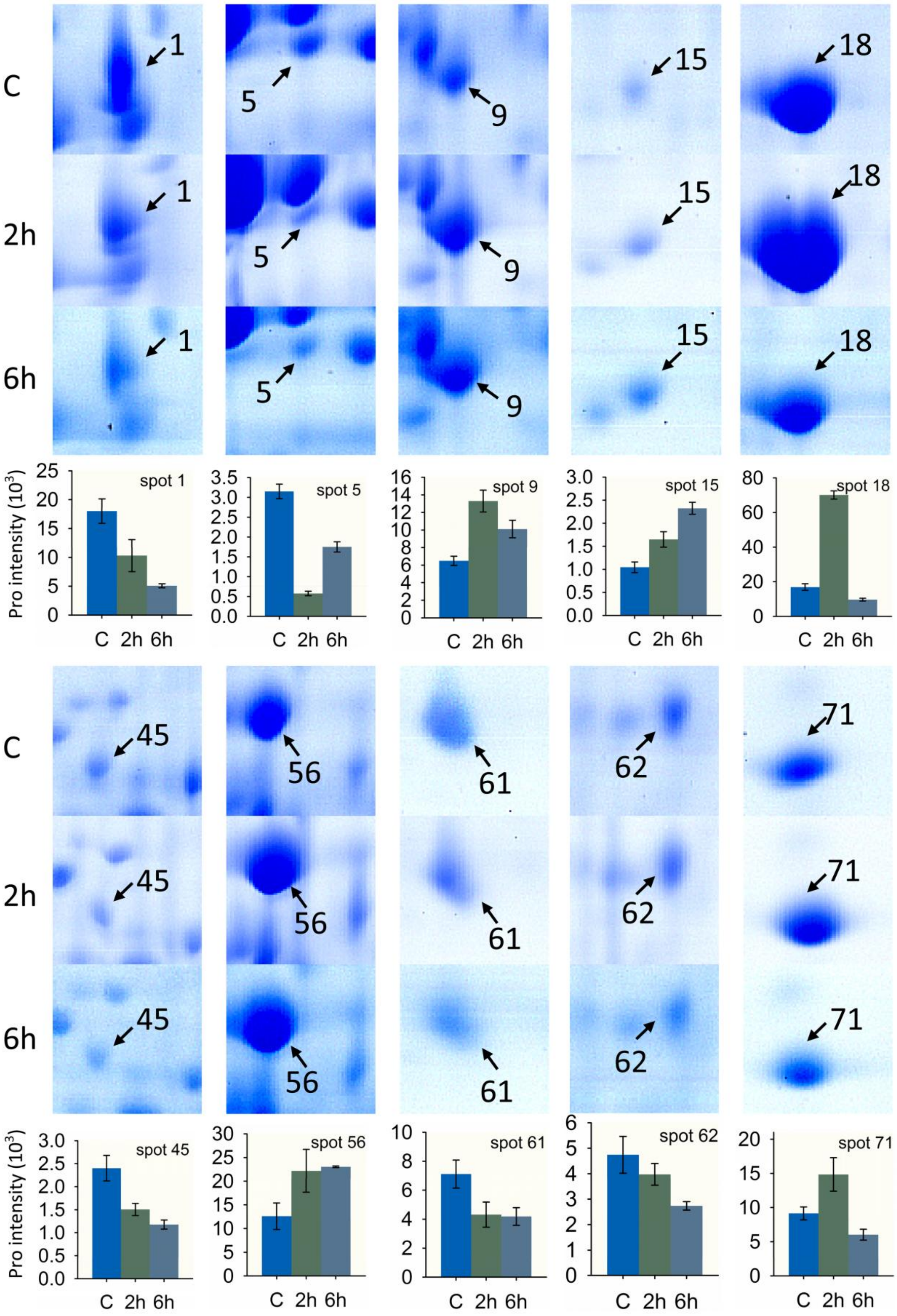

Supplementary Figure S3. Typical examples of DEPs showing different profiles. Ten protein spots are enlarged from Figure 3 and protein intensity (pro intensity) values are from Supplementary Table S1. C, 2 h, and 6 h represent control, 2 h, and 6 h after treatment, respectively. Error bars means ±SD of three independent biological replicates.
